# Supplementary material for: The Effect of Microbial Diversity and Biomass on Microbial Respiration in Two Soils along the Soil Chronosequence
Source: Microorganisms. 2022 Sep 27;10(10):1920. doi: 10.3390/microorganisms10101920 (PMC9609397; doi:10.3390/microorganisms10101920)
Supplement: Supplementary file 1 [file microorganisms-10-01920-s001.zip › microorganisms-1910307-supplementary.pdf]

Supplementary Materials

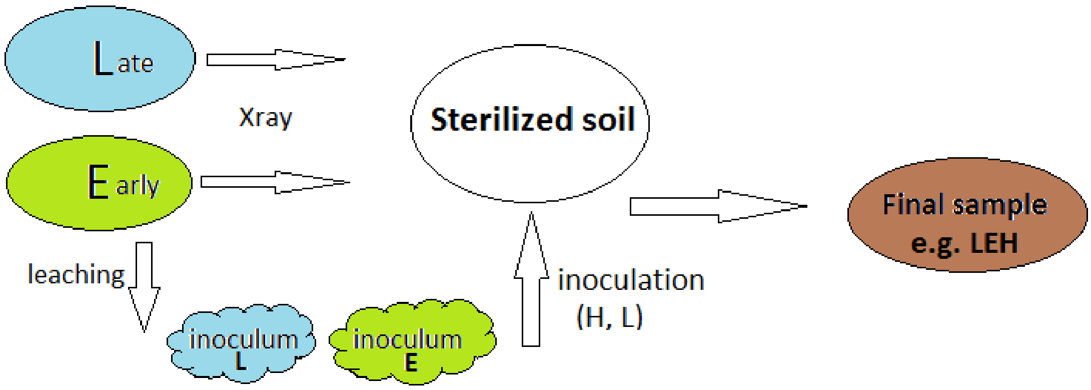

Figure S1. The experimental design. H or L means inoculation diversity, which is negatively related to dilution. H means high diversity (less diluted) inoculum, and L means less diverse (more diluted) inoculum.
